# Effective Mechanical Advantage About the Ankle Joint and the Effect of Achilles Tendon Curvature During Toe-Walking

**DOI:** 10.3389/fphys.2020.00407

**Published:** 2020-05-19

**Authors:** Carla Harkness-Armstrong, Héloïse A. Debelle, Constantinos N. Maganaris, Roger Walton, David M. Wright, Alfie Bass, Vasilios Baltzopoulos, Thomas D. O’Brien

**Affiliations:** ^1^Research Institute for Sport and Exercise Sciences, Liverpool John Moores University, Liverpool, United Kingdom; ^2^Alder Hey Children’s NHS Foundation Trust, Liverpool, United Kingdom

**Keywords:** mechanical advantage, moment arm, Achilles tendon, equinus, ultrasound

## Abstract

**Aim:** To study the causes of locomotor dysfunction, estimate muscle forces, or understand the influence of altered sarcomere and muscle properties and behaviours on whole body function, it is necessary to examine the leverage with which contractile forces operate. At the ankle joint, current methods to quantify this leverage for the plantarflexors do not account for curvature of the Achilles tendon, and so may not be appropriate when studying equinus gait. Thus, novel methodologies need to be developed and implemented to quantify the Achilles tendon moment arm length during locomotion.

**Methods:** Plantarflexor internal moment arm length and effective mechanical advantage of 11 typically developed young adults were calculated throughout stance, while heel-toe walking and voluntarily toe-walking on an instrumented treadmill. Achilles tendon moment arm was defined in two-ways: (1) assuming a straight tendon, defined between the gastrocnemius medialis myotendinous junction and Achilles tendon insertion point, and (2) accounting for tendon curvature, by tracking the initial path of the Achilles tendon from the calcaneal insertion.

**Results:** When accounting for tendon curvature, Achilles tendon moment arm length and plantarflexor effective mechanical advantage did not differ between walking conditions (*p* > 0.05). In contrast, when assuming a straight tendon, Achilles tendon moment arm length (*p* = 0.043) and plantarflexor effective mechanical advantage (*p* = 0.007) were significantly greater when voluntary toe-walking than heel-toe walking in late stance.

**Discussion:** Assuming a straight Achilles tendon led to a greater Achilles tendon moment arm length and plantarflexor effective mechanical advantage during late stance, compared to accounting for tendon curvature. Consequently, plantarflexor muscle force would appear smaller when assuming a straight tendon. This could lead to erroneous interpretations of muscular function and fascicle force-length-velocity behaviour *in vivo*, and potentially inappropriate and ineffective clinical interventions for equinus gait.

## Introduction

In human locomotion, propelling the body forward necessitates the development of joint moments which exceed those caused by the ground reaction force (GRF). To achieve this, the involved muscles must produce adequate contractile force. In addition to the capacity to voluntarily activate the contracting muscles, the length and velocity at which these muscles contract during stance is vital, as these parameters will dictate contractile force output according to the force-length-velocity relationship (Gordon et al., 1966). During walking, the gastrocnemius medialis muscle of typically developed adults has been shown to operate quasi-isometrically and close to the region where optimal sarcomere overlap would be expected (Fukunaga et al., 2001). This behaviour favours the economical production of high contractile forces. However, certain neuromuscular pathologies, such as cerebral palsy (CP) can negatively affect this optimal behaviour. Differences in sarcomere length and function (Ponten et al., 2007; Lieber et al., 2017), muscle stiffness (Barber et al., 2011), and lengthening characteristics (Kalkman et al., 2018) have been documented in those with CP, and associated differences in fascicle behaviour during gait observed (Barber et al., 2017). These may explain why these children find it difficult to generate the required forces for adequate moment development about the ankle joint.

However, to study the causes of locomotor dysfunction, estimate muscle forces, or understand the influence of altered sarcomere and muscle properties and behaviours on whole body function, it is also necessary to examine the leverage with which the contractile forces operate. At the ankle, the ratio between the internal moment arm of the plantarflexors and the external moment arm of the GRF, also known as effective mechanical advantage, will determine the required muscle forces for gait. Alterations in either moment arm can greatly affect the required muscle forces. Therefore, understanding how this leverage may be impairing muscle function and/or contributing to movement impairments is vital in order to provide the appropriate interventions to improve mobility.

It is well known that children with CP have alterations in both the internal and external moment arms, due to skeletal deformities and atypical gait patterns (Theologis, 2013). Deformity of the hindfoot can alter the internal muscle-tendon moment arm, which may be either increased (Alexander et al., 2019) or decreased (Kalkman et al., 2017) according to previous studies of the ankle joint at rest. Such alterations can affect the production of internal joint moments due to a change in the required muscle force (Gage et al., 2009). Dysfunction of the external GRF moment arm can arise from foot deformities or atypical gait patterns, such as a midfoot break or equinus (Theologis, 2013). However, atypical gait patterns may also be compensatory behaviours for muscle weakness or alterations in the internal moment arm, because shortening the distance between the ankle joint axis and the GRF vector will reduce the joint moment that must be overcome (Hampton et al., 2003).

To treat lever arm dysfunction, surgical (Firth et al., 2013; Chung et al., 2015) or therapeutic interventions such as Botulinum toxin-A injections or serial casting (MacKay-Lyons et al., 2017; Multani et al., 2019) are used. Such interventions alter the effective mechanical advantage of the plantarflexors and consequently, the muscle force requirements. Despite this, effective mechanical advantage is rarely ever quantified in research or even considered in clinical decision making for a group of children that commonly experience muscle weakness (Hampton et al., 2003). Therefore, the effects of these interventions on muscle function in children with CP are not well understood.

The lack of experimental evidence and clinical application may be because quantifying effective mechanical advantage, and specifically the Achilles tendon moment arm length, during functional tasks is complex. Only two known studies have implemented direct measurements of the Achilles tendon moment arm during gait (Rasske et al., 2017; Rasske and Franz, 2018). They combined motion capture with tracked ultrasound images of the proximal region of the Achilles tendon. The Achilles tendon line of action is extrapolated distally from a locally visible portion of the tendon within the ultrasound viewing window, to estimate moment arm length about the ankle joint axis. If the tendon distally to the scanned region remains straight over the same action line, then the moment arm length quantified by the linear extrapolation is an accurate representation of the force vector acting on the calcaneus during plantarflexor muscle contraction to generate ankle joint rotation. However, it is known that the Achilles tendon becomes curved in plantarflexed positions (Obst et al., 2014), due to its geometric architecture and configuration with surrounding tissues (Kinugasa et al., 2018). Such curvature would alter the orientation of the force acting on the calcaneus, bringing the vector closer to the ankle joint axis, thus reducing moment arm length (Csapo et al., 2013; Kinugasa et al., 2018). Therefore, defining the Achilles tendon line of force to act over a straight line, defined from any portion of the tendon more proximal than the curvature, grossly simplifies the anatomy and mechanics of the joint and may be subject to errors in estimating the moment arm length over the tendon’s distal region. This is particularly relevant when studying equinus gait patterns where large plantarflexion angles occur.

The only known study (Obst et al., 2017) to have accounted for tendon curvature when calculating Achilles tendon moment arm found that during passive joint rotations, assuming a straight path of the tendon overestimated moment arm length in plantarflexed positions, compared to accounting for curvature. However, this study used a 3D ultrasound sweep to define the curved action line of the Achilles tendon (Hashizume et al., 2012), which would not be possible in functional tasks such as gait, and it is unknown if the conclusion holds under dynamic loaded conditions. The alternative, of adapting the previous dynamic ultrasound method (Rasske et al., 2017; Rasske and Franz, 2018) to track the distal free tendon is not possible in equinus gait, as the ultrasound probe cannot maintain contact with the skin and becomes unstable.

Thus, to improve our understanding of coordinated bodily movement and estimate required contractile forces, novel methodologies need to be developed and implemented to quantify the Achilles tendon moment arm length during locomotion. Therefore, this study developed a practical method to quantify the Achilles tendon moment arm and account for curvature which is applicable to *in vivo* locomotor tasks, such as gait. The aim was to calculate the effective mechanical advantage of the plantarflexors during heel-toe walking and voluntary toe-walking gait and assess whether accounting for Achilles tendon curvature would alter effective mechanical advantage comparisons between walking conditions, when compared to a straight Achilles tendon. We hypothesised that Achilles tendon moment arm length would be smaller when accounting for tendon curvature. Therefore, we also hypothesised that the increase in effective mechanical advantage when voluntary toe-walking compared to heel-toe walking would be smaller when accounting for tendon curvature, compared to a straight Achilles tendon.

## Materials and Methods

### Participants

Eleven typically developed young adults (male *n* = 4; female *n* = 7; age 24 ± 3 years; height 1.76 ± 0.05 m; body mass 73 ± 6 kg) were recruited for this study. To assess test-retest reliability, seven participants (male *n* = 1; female *n* = 6; age 25 ± 3 years; height 1.75 ± 0.06 m; body mass 74 ± 6 kg) returned later within the same day to repeat the protocol. All participants were free from lower-limb injuries within the 6 months prior to the study. Written informed consent was obtained and the study was conducted in accordance with the recommendations of the institutional ethics committee and the declaration of Helsinki.

### Measurement Protocol

**FIGURE 1:**
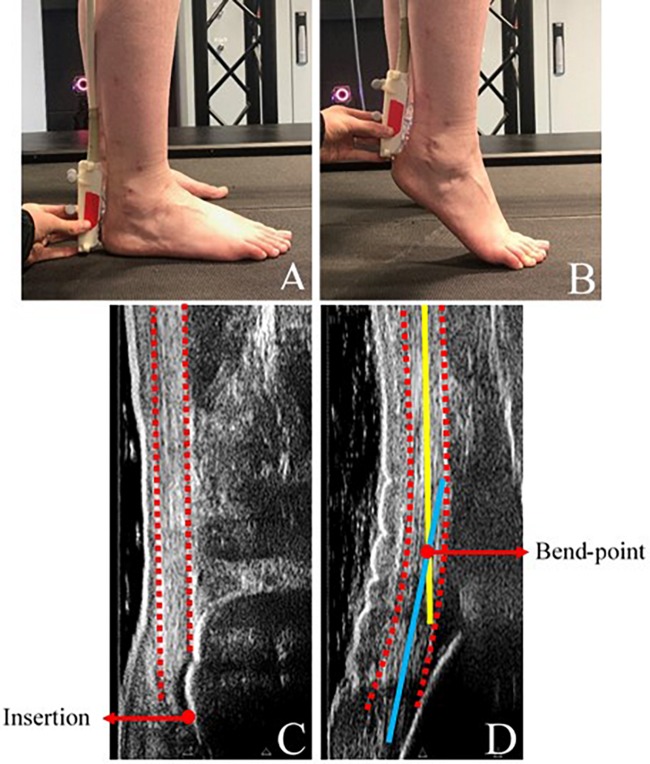
**(A)** Experimental set-up, showing ultrasonography positioned in the sagittal plane to identify Achilles tendon insertion and **(B)** Achilles tendon bend-point. **(C)** Identification of Achilles tendon insertion from ultrasound images taken during standing. **(D)** Identification of Achilles tendon bend-point from ultrasound images taken during loaded plantarflexion. Dashed red: Outline of Achilles tendon path, solid yellow: straight portion of Achilles tendon, solid blue: initial path of Achilles tendon. Bend-point was defined as the intersection of the yellow and blue lines. During data collection, the bend-point was judged visually.

**FIGURE 2:**
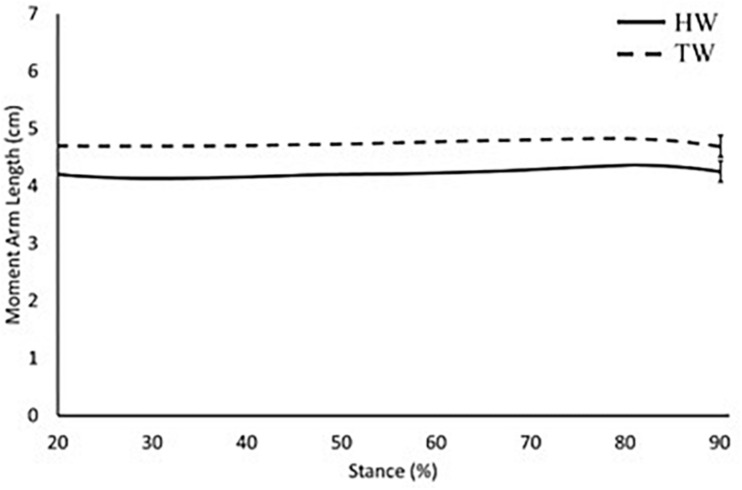
Internal moment arm length across 20–90% of stance when accounting for Achilles tendon curvature. Solid black: heel-toe walking, dashed black: voluntary toe-walking. For clarity, only average standard error of the mean bars are indicated.

To identify the anatomical features of the Achilles tendon, static ultrasound images were obtained in the sagittal plane whilst participants stood under their own body weight (1) with feet-flat on the floor (Figure 1A), to locate Achilles tendon insertion (Figure 1C), and (2) on toes (Figure 1B), to identify the Achilles tendon bend point in plantarflexed positions (Figure 1D) (60 mm linear B-mode transducer; Telemed Echoblaster, Vilnius, Lithuania). Achilles tendon bend-point was judged visually as the point of intersection between the initial path of the tendon from the calcaneal insertion, and the main portion of the tendon (Figure 1D). Both insertion and bend-point were marked on the skin with surgical marker.

Passive retro-reflective markers were placed on bony anatomical landmarks of the right foot and shank, including medial/lateral femoral epicondyles, medial/lateral malleoli, and 1st, 2nd, and 5th metatarsal heads. To track the Achilles tendon path into the calcaneal insertion, the calcaneal marker was placed directly on the tendon insertion skin marker. A second marker was placed distally to the Achilles tendon bend-point skin marker, to ensure that the marker remained below the defined bend-point. To track the myotendinous junction (MTJ) location, for the purpose of defining a straight Achilles tendon, the ultrasound scanner was securely fastened over the MTJ of the medial gastrocnemius and Achilles tendon using a custom-made probe holder, rigidly fitted with a cluster of three markers. To minimise out of plane movement, the long axis of the transducer was aligned with the line of action of the muscle.

Participants walked on an instrumented split-belt treadmill (Motek Medical, Amsterdam, Netherlands) at 1.2 m.s^–1^ in two walking conditions; (1) typical heel-toe gait, and (2) voluntary toe-walking, whilst secured in an upper body fall-arrest harness for safety. Before data were recorded, 5 mins of familiarisation was provided. At completion of the familiarisation period, participants continued to walk for a further five gait cycles, during which three-dimensional kinematics were collected using a 12-camera Vicon Vero system (Vicon, Oxford, United Kingdom), at a sample rate of 120 Hz. Force data were also recorded within Vicon at a sample rate of 1200 Hz. Ultrasound data were recorded at 30 Hz, synchronised to motion data by a 5 V digital signal captured in Vicon. All data for both methods of defining Achilles tendon line of action were collected simultaneously. For test-retest measurements, all equipment was fully removed and relocated.

### Data Processing

Kinematic and kinetic data were processed in Visual 3D software (C-Motion, Rockville, MD, United States). All data were low-pass filtered with a cut-off frequency of 6Hz. Stance phase was defined between initial contact and toe-off of the right foot, identified using a force plate threshold of 50N. Data were then cropped between 20 and 90% of stance to only include positive external moment arm values. Ultrasound images of calcaneus and Achilles tendon bend-point, and videos of MTJ displacement were analysed manually in ImageJ software (ImageJ 1.51j8, United States). All data were exported to Matlab (MathWorks R2019a, United Kingdom), where subsequent analysis was conducted using a custom-made script.

Effective mechanical advantage (EMA) was calculated between 20 and 90% of stance using Eq. (1):

(1) $$\begin{array}{c} E\,M\,A=\frac{I\,n\,M\,A}{E\,x\,M\,A} \end{array}$$

where, *InMA* is the internal moment arm length and *ExMA* is the external moment arm length.

External moment arm length was calculated as the perpendicular distance between the *trans*-malleolar axis, defined as a straight line between medial and lateral malleoli markers, and the GRF vector.

Internal moment arm length was calculated as the perpendicular distance between the *trans*-malleolar axis and the line of action of the Achilles tendon force. To define the Achilles tendon action line, the calcaneal and bend-point markers were first corrected anteriorly along the foot plane by the respective distances measured within the static ultrasound images (Figures 1C,D), to lie over the mid-point of the tendon. Achilles tendon line of action was then defined as a straight line between the Achilles tendon insertion and (1) the MTJ, to assume a straight tendon, and (2) the corrected Achilles tendon bend-point marker, to account for tendon curvature. Data were averaged for all five strides which were collected per participant.

### Statistics

All parameters were normally distributed. To test whether the internal moment arm length and effective mechanical advantage differed between walking conditions or analysis methods, two one-way ANOVAs were performed using Statistical Parametric Mapping (SPM1D). Analyses were completed in Matlab 2019a, with additional *post hoc* tests, and significance set at *p* < 0.05. An SPM1D paired *t*-test was used to determine whether the external moment arm length differed between walking conditions only, as differences between analyses methods were eliminated by collecting data simultaneously. The intra-rater reliability was assessed by calculating the average mean typical error throughout stance between test-retest sessions.

## Results

**FIGURE 3:**
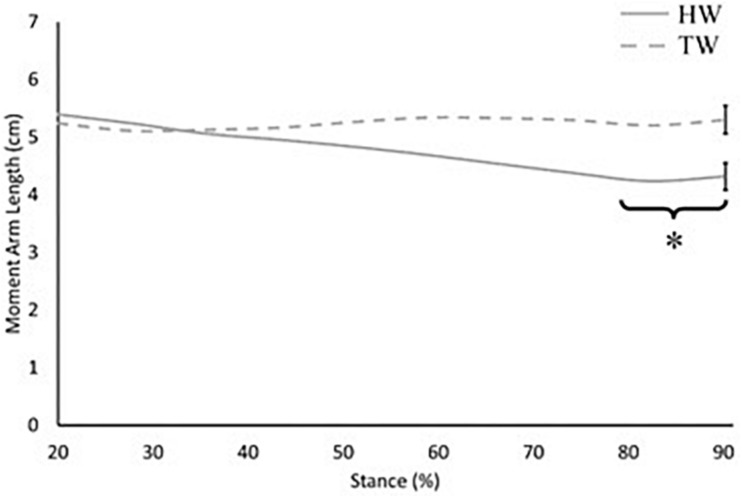
Internal moment arm length across 20–90% of stance when assuming a straight Achilles tendon. Solid grey: heel-toe walking (HW), dashed grey: voluntary toe-walking (TW). *Significant difference between HW and TW from 80% of stance onward (*p* = 0.043). For clarity, only average standard error of the mean bars are indicated.

When accounting for Achilles tendon curvature, internal moment arm length was constant throughout stance, with no significant difference between heel-toe walking and voluntary toe-walking conditions (mean 4.6 vs. 4.7 cm, *p* > 0.05; Figure 2). When assuming a straight Achilles tendon, internal moment arm length was also constant throughout stance in the voluntary toe-walking condition (mean 5.2 cm) but decreased throughout stance in the heel-toe walking condition. This led to a significantly smaller internal moment arm length in the heel-toe walking condition than in voluntary toe-walking from 80% of stance onward (∼4.4 vs. ∼5.3 cm, *p* = 0.043; Figure 3).

**FIGURE 4:**
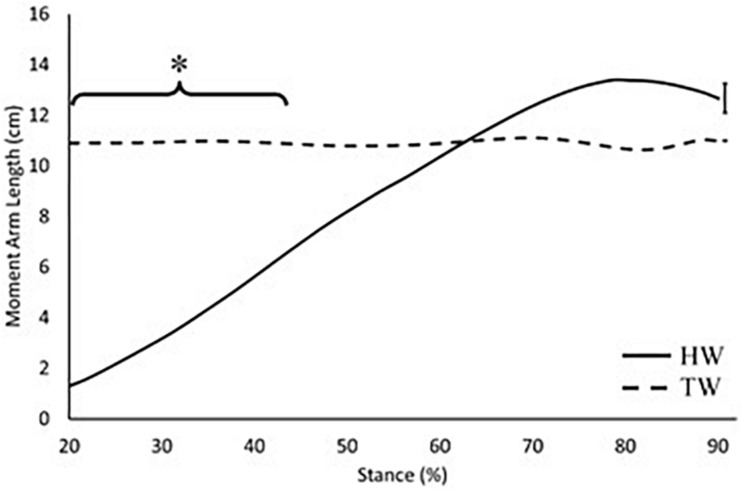
External moment arm length across 20–90% of stance. Solid black: heel-toe walking, dashed black: voluntary toe-walking. *Significant difference between HW and TW between 20 and 40% of stance (*p* < 0.001). For clarity, only average standard error of the mean bars are indicated.

External moment arm length in the voluntary toe-walking condition remained relatively constant throughout stance (mean 11 cm), whereas for the heel-toe walking condition, external moment arm length increased throughout stance (Figure 4). External moment arm length was significantly smaller in the heel-toe walking condition between 20 and 40% of stance (*p* < 0.001; Figure 4), whereas there was no difference between conditions during late stance, where large plantarflexion moments are produced.

**FIGURE 5:**
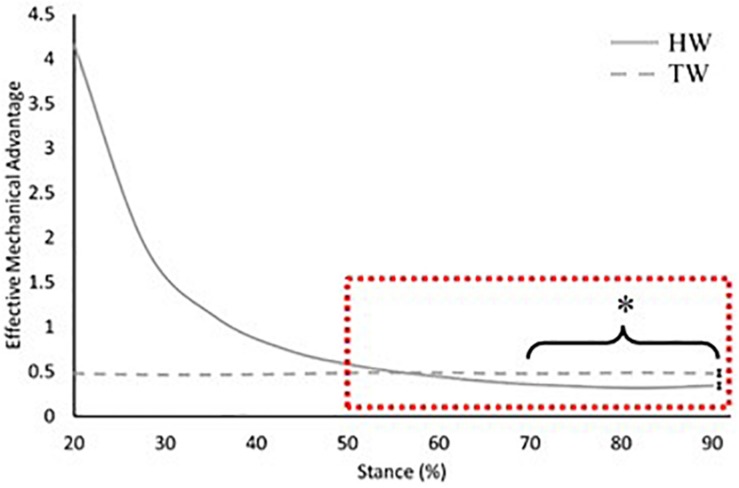
Effective mechanical advantage of the ankle joint across 20–90% of stance when assuming a straight Achilles tendon. Solid grey: heel-toe walking (HW), dashed grey: voluntary toe-walking (TW), dashed red box: region of interest for statistical comparisons. *Significant difference between HW and TW from 70% of stance onward (*p* = 0.007). For clarity, only average standard error of the mean bars for the region of interest are indicated.

**FIGURE 6:**
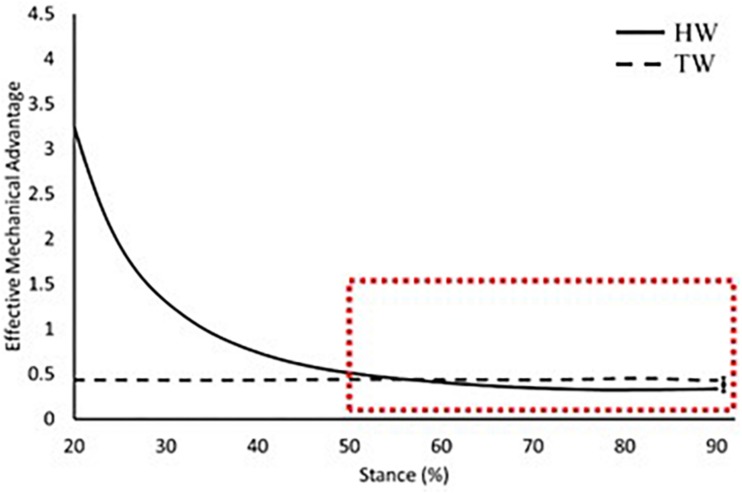
Effective mechanical advantage of the ankle joint across 20–90% of stance when accounting for Achilles tendon curvature. Solid black: heel-toe walking, dashed black: voluntary toe-walking, dashed red box: region of interest for statistical comparisons. For clarity, only average standard error of the mean bars for the region of interest are indicated.

When assuming a straight tendon, effective mechanical advantage was significantly smaller in the heel-toe walking condition compared to the voluntary toe-walking condition from 70% of stance onward (average mean difference = 0.2, *p* = 0.007; Figure 5). Whereas, there was no difference in the effective mechanical advantage between walking conditions when accounting for Achilles tendon curvature (average mean difference = 0.1, *p* > 0.05; Figure 6).

Both methods of defining Achilles tendon line of action showed good intra-rater test-retest reliability in both walking conditions, with an average mean typical error of effective mechanical advantage throughout stance of 0.05 (coefficient of variation = 4%).

## Discussion

In this study, we have developed a reliable method to account for Achilles tendon curvature in the assessment of internal moment arm length and effective mechanical advantage of the plantarflexors during locomotion. Using this method, we found significantly smaller estimates of the plantarflexors’ mechanical advantage compared to methods assuming a straight Achilles tendon, particularly in plantarflexed positions. This will have implications for the estimation of the contractile forces required from the plantarflexors during gait to propel the body forward.

Assuming a straight tendon led to a larger Achilles tendon moment arm length during locomotion compared to accounting for tendon curvature. This observation is consistent with previous measurements from passive ankle joint rotations (Obst et al., 2017). Regardless of walking condition, Achilles tendon moment arm length was greater throughout stance when assuming a straight Achilles tendon, with the greatest differences apparent in voluntary toe-walking, where plantarflexion angle and thus tendon curvature is greatest (Kinugasa et al., 2018). These findings are also reflected in the effective mechanical advantage comparisons, as differences in the external moment arm lengths were eliminated between analysis methods. Consequently, a larger Achilles tendon moment arm length, and therefore effective mechanical advantage, would result in smaller estimations of required plantarflexor force for locomotion. Thus, different conclusions could be reached depending on the method used to define the Achilles tendon line of action. The magnitude of this difference would be further increased if comparing between plantarflexed and more neutral joint angles, for example, between toe and heel-toe walking.

The decrease found in the Achilles tendon moment arm length throughout stance when assuming a straight Achilles tendon is in contrast with previous work. Rasske et al. (2017) reported slight increases in Achilles tendon moment arm length from 4 to 4.3 cm throughout stance when assuming a straight Achilles tendon. The observed differences in the direction of this change could be due to methodological differences in defining the ankle axis and Achilles tendon line of action, for the calculation of moment arm length. Rasske et al. (2017) considered the moment arm to be between the Achilles action line and the ankle joint centre. In contrast, we calculated the moment arm as the shortest distance to the vector along the *trans*-malleolar axis. Furthermore, Rasske et al. (2017) tracked a section of the Achilles tendon more distal than the gastrocnemius medialis MTJ, but proximal to the curvature, while our “straight” method defined the tendon between the MTJ and calcaneal insertion, to reflect the overall path of the muscle-tendon unit between the mechanically important regions for proximal to distal force transmission, from muscle to tendon, and from tendon to bone. Nevertheless, similar Achilles tendon moment arm lengths were found in late stance (4.3 cm), where meaningful plantarflexion moments are produced.

The external GRF moment arm length increased throughout stance in the heel-toe walking condition (Figure 4), which is consistent with previous research (Giddings et al., 2000). This is due to the progression of the GRF vector along the foot and is the main reason why internal plantarflexion moments increase throughout stance. Since this magnitude of change is much greater than the changes in the internal moment arm length, the external moment arm plays the major role in determining the profile of effective mechanical advantage throughout stance. When voluntary toe-walking, there was no progression of the GRF vector along the foot, thus the external moment arm length remained relatively constant throughout stance (Range: 10.6–11.1 cm). This can therefore explain why the profile of the effective mechanical advantage also remains constant within this condition (Figures 5, 6).

We compared the effective mechanical advantage between walking conditions in late stance, where meaningful plantarflexion moments are produced in heel-toe walking. Differences in the external moment arm length between analysis methods were eliminated, therefore comparisons of the effective mechanical advantage between walking conditions are explained by the differences found in the Achilles tendon moment arm length. When accounting for tendon curvature, there was no difference in the effective mechanical advantage between walking conditions (Figure 6), whereas assuming a straight Achilles tendon led to a greater effective mechanical advantage in voluntary toe-walking compared to heel-toe walking from 70% of stance onward (Figure 5). Consequently, assuming a straight Achilles tendon would result in smaller estimates of the required plantarflexor muscle force for propulsion, compared to accounting for tendon curvature. For clinical context, the discrepancy in required muscle force at peak plantarflexion moment was less than 5% for heel-toe walking. Therefore, when assessing heel-toe walking only, a straight tendon path method may be acceptable, as the combination of a relatively small plantarflexion angle and large plantarflexor moment is unlikely to cause large curvature of the tendon. However, when assessing toe-walking, where large curvature of the tendon is expected, the discrepancy in required muscle force was ∼10%. Therefore, adopting a straight tendon path method may not be appropriate for toe-walking, particularly when comparing pre and post clinical interventions whereby walking pattern, and potentially tendon curvature, will be altered.

Data from the present study indicate that equinus gait increases the effective mechanical advantage of the plantarflexors from 60% of stance onward (Figures 5, 6), the period when meaningful plantarflexor moments are produced to, in part, propel the body forward. Therefore, the required muscle force would be reduced, supporting the notion that equinus gait would serve as a compensatory strategy to accommodate plantarflexor weakness (Hampton et al., 2003). Many clinical interventions for children with CP aim to improve walking pattern by altering the ankle joint range of motion or lengthening the external moment arm along the foot (Firth et al., 2013; Chung et al., 2015; MacKay-Lyons et al., 2017; Multani et al., 2019). Both would impact the effective mechanical advantage of the plantarflexors, yet little is known about the impact on muscle function. Surgical lengthening of the Achilles tendon in particular may also result in changes to the Achilles tendon curvature, due to alterations in gross muscle-tendon architecture and the relationship with surrounding soft tissue structures. Nevertheless, we have shown that different conclusions could be reached when comparing heel-toe walking to voluntary toe-walking, depending on how the Achilles tendon line of action was defined. Therefore, consideration of these erroneous effective mechanical advantage values in the decision-making process for management and treatment of equinus may lead to ineffective or inappropriate clinical interventions. For example, an overestimation of the effective mechanical advantage would suggest that a child has adequate plantarflexor strength to meet the demands of locomotion post-intervention, when muscle force was initially underestimated. Thus, the ability to measure the effective mechanical advantage correctly is essential if we are to start incorporating such measures into clinical practice. Musculoskeletal modelling offers an alternative to direct measurement but may not be appropriate, or routinely feasible due to the challenges of including individualised skeletal deformities for children with CP (Rezgui et al., 2013). As a result, direct measurements are needed in order to start providing individualised conclusions and recommendations for these children.

In this study, we have shown a need to account for tendon curvature when assessing equinus gait. However, further work is required to systematically study the proposed method, sources of error and anatomical validity in both typically developed and pathological populations. For example, the extent to which the tendon bend-point changes under altered joint angle and loading with respect to skin-mounted markers remains unclear. A number of limitations should be acknowledged. First, we defined the ankle joint axis of rotation using the *trans*-malleolar axis rather than the true ankle axis of rotation, which operates around the talo-crural joint, with a varying orientation across the ankle joint range of motion (Barnett and Napier, 1957; Lundberg et al., 1989). The use of functional joint calibrations may provide closer estimates (Wade et al., 2019). However, our method was developed for application in patients with gait pathologies and skeletal deformities, for whom functional joint calibrations may not be possible. A *trans*-malleolar axis would also account for bony deformities which are often present in conditions such as CP.

Marker placement error and skin movement artefact may have introduced error in the tracking of the *trans*-malleolar axis and the Achilles tendon (Peters et al., 2010). Although we took care in acquisition of static ultrasound images, we cannot rule out that aligned and pixelation errors (Goldstein, 2000) impacted the correction of motion capture markers. However, we have shown good intra-rater reliability between test-retest sessions, with a mean typical error which is smaller than the magnitude of the differences between our measurements. Finally, the present study has simulated toe-walking in a group of typically developed young adults. Previous work has highlighted differences in the muscle behaviour of children with CP who walk in equinus and children and adults who voluntarily toe-walk (Kalsi et al., 2016), therefore further work will be conducted to assess the applicability of this method in children with gait pathologies.

To conclude, we have shown that it is essential to account for Achilles tendon curvature in plantarflexed ankle positions, as assuming a straight Achilles tendon resulted in a larger Achilles tendon moment arm length and therefore effective mechanical advantage. Consequently, the required muscle force would be smaller when assuming a straight tendon, which could lead to erroneous interpretations of muscular function and sarcomere force-length-velocity behaviour *in vivo*, and potentially inappropriate and ineffective clinical interventions. We have shown that Achilles tendon curvature can be accounted for *in vivo* during functional tasks such as gait using a relatively simple method, which could facilitate the implementation of measurements of effective mechanical advantage into clinical practice. This would allow for future work to assess how clinical interventions alter the effective mechanical advantage, and the resultant impact on muscle function in children with equinus gait. However, first, further work is required to validate the proposed method and assess the applicability in children with gait pathologies.

## Data Availability Statement

The datasets generated for this study are available on request to the corresponding author.

## Ethics Statement

The studies involving human participants were reviewed and approved by Liverpool John Moores University Research Ethics Committee. The patients/participants provided their written informed consent to participate in this study, in accordance with the Declaration of Helsinki.

## Author Contributions

CH-A, TO’B, CM, and VB contributed to conception and design of the research. CH-A and HD acquired the data. CH-A analysed the data and drafted the manuscript. All authors interpreted the results, edited and revised the manuscript and agreed to its submission for publication.

## Conflict of Interest

The authors declare that the research was conducted in the absence of any commercial or financial relationships that could be construed as a potential conflict of interest.
